# The Association of *Achromobacter xylosoxidans* Airway Infection with Disease Severity in Cystic Fibrosis

**DOI:** 10.3390/jcm14072437

**Published:** 2025-04-03

**Authors:** Ophir Bar-On, Meir Mei-Zahav, Hagit Levine, Huda Mussaffi, Hannah Blau, Haim Ben Zvi, Dario Prais, Patrick Stafler

**Affiliations:** 1 Graub CF Center, Pulmonary Institute, Schneider Children’s Medical Center of Israel, Petach Tikva 4920235, Israel; ophirbo@gmail.com (O.B.-O.); mmeizahav@gmail.com (M.M.-Z.); hagitlevine@gmail.com (H.L.); hgeorgy@gmail.com (H.M.); hannahblau@hotmail.com (H.B.); darioprais@gmail.com (D.P.); 2 School of Medicine, Faculty of Medical and Health Sciences, Tel-Aviv University, Tel Aviv 6997801, Israel; haimbe@clalit.org.il; 3 Clinical Microbiology Laboratory, Rabin Medical Center, Beilinson Hospital, Petah Tikva 4941492, Israel

**Keywords:** Cystic Fibrosis, *Achromobacter xylosoxidans*, chronic infection, lung function

## Abstract

**Background/Objectives**: The prevalence of *Achromobacter xylosoxidans* is increasing in people with Cystic Fibrosis (pwCF), yet its clinical pathogenicity remains controversial. The objective of this study was to chart the longitudinal prevalence and examine clinical associations before and after infection. **Methods**: This observational, retrospective study was conducted at a single CF center over a 14-year period. Data were collated from patient charts and clinic databases. Patients with *Achromobacter* sputum cultures were compared to those without the bacterium and analyzed according to whether they had single, intermittent, or chronic infections. **Results**: During the study period, an annual average of 124 pwCF were followed up at our clinic, with a median age of 13.6 years (IQR = 7.6–27.7). The *Achromobacter* detection rate increased from 0 to 6.1%. Twenty-three percent (29/124) of patients had at least one positive culture. The median age at acquisition was 17 years (IQR = 14.5–33). At the time of acquisition, the median FEV_1_ was 81% (IQR = 46–94), compared to 90% (IQR = 72–99) for patients without *Achromobacter*, *p* < 0.001. Patients with *Achromobacter* tended to demonstrate more chronic *Pseudomonas* (55% vs. 27%, *p* = 0.06) and pancreatic insufficiency (66% vs. 47%, *p* = 0.07). At two years post-acquisition, the median FEV_1_ for patients with intermittent and chronically infected decreased by 11.5% (IQR = −3.75–7.5), compared to 1.5% (IQR = −2.5–12.5) for those with a single positive culture, *p* = 0.03. Similarly, pulmonary exacerbations per year became more frequent post-acquisition in intermittent and chronically infected patients: Median (range) 2.5 (0–8) pre-, versus 3.0 (0–9) post-acquisition, *p* = 0.036. **Conclusions**: Chronic and intermittent infection with *Achromobacter* were associated with accelerated lung function decline and increased exacerbation frequency. Larger prospective studies are needed to confirm these findings and examine the effect of eradication on the clinical course.

## 1. Introduction

Bacterial airway infections continue to be the main cause of morbidity and mortality in Cystic Fibrosis (CF) [1]. The most common pathogens are *Pseudomonas aeruginosa* (*P. aeruginosa*), *Staphylococcus aureus*, and *Haemophilus influenza*. But as life expectancy in individuals with CF rises, the complexity of airway infections increases, with the emergence of further bacterial pathogens, including *Burkholderia*, *Stenotrophomonas*, *non-tuberculous mycobacteria*, *Achromobacter*, and others [2].

*Achromobacter xylosoxidans* (*A. xylosoxidans*) is increasingly isolated from the sputum of people with CF (pwCF), with prevalence ranging from 2.3% to 29.3% in different centers around the world [3,4,5,6]. The European CF registry only started monitoring *A. xylosoxidans* infection in 2018 [7] and reported an average prevalence of 3.75% across Europe, while the 2019 United States CF registry reported a 6.1% prevalence [8]. These figures are likely an underestimation, as taxonomy has been evolving over the years [9] due to difficulties with exact laboratory identification [10]; for instance, *A. xylosoxidans* used to be confused with other Gram-negative bacteria, especially *P. aeruginosa* [11].

Recent in vitro and animal studies have shown that *A. xylosoxidans* shares important pathophysiological features with better-known CF pathogens, likely creating a competitive survival advantage in the ecological niche of the CF lung and contributing to the vicious circle of infection and inflammation [12,13].

Nevertheless, the clinical impact of *A. xylosoxidans* infection remains controversial. Some reports indicate no effect on lung function [3,4,14], while others have demonstrated a more rapid decline in lung function following infection, as well as more frequent exacerbations, including fatal ones [4,5,15]. A recent analysis of clinical outcomes associated with *Achromobacter species* from the European CF Society Patient Registry also suggested association with disease severity, of similar magnitude to infection with *P. aeruginosa* [16].

The aims of our study were to describe the longitudinal prevalence of *A. xylosoxidans* infection in our CF cohort and evaluate the clinical status of pwCF with *A. xylosoxidans* airway infection before and after its acquisition. Of note, our observation period occurred prior to the widespread use of highly effective CFTR modulators.

## 2. Methods

### 2.1. Study Design and Population

This observational, longitudinal, retrospective study included all patients with CF attending the Graub CF Center at Schneider Children’s Medical Center of Israel from January 2007 to December 2020. During this period, the clinic population consisted of both pediatric and adult patients. Subjects were diagnosed with CF according to accepted criteria [17]. Data were retrieved from charts of patients with CF and the annual CF clinic database. Patients who ever had any sputum cultures positive for *A. xylosoxidans* during the observation period were compared with those who never grew *A. xylosoxidans*. Parameters examined included age, gender, CFTR mutation class, forced expiratory volume in 1 s, percent of predicted (FEV_1_%pp), *Pseudomonas* status, body mass index (BMI), pancreatic enzyme replacement therapy, the presence of CF-related diabetes (CFRD), and intravenous (IV) antibiotic courses administered.

Data for pwCF with *A. xylosoxidans* in sputum cultures were analyzed in more detail, comparing the period of two years prior to two years after acquisition. For the sake of comparison, data for pwCF with sputum cultures negative for *A. xylosoxidans* were taken from the annual clinic CF database of 2014, the mid-point of data collection.

### 2.2. Definitions

We adapted the European CF Society patient registry’s definition of chronic infection [7]: “Patient should be regarded as chronically infected if they fulfill the criteria now (or in recent years and the status is expected to be preserved), when a. >50% of respiratory samples during the last 12 months are positive; at least 4 samples during that period (modified Leeds criteria) are positive; and/or b. significantly raised bacteria-specific antibodies are present”. To accommodate the longitudinal design of our study, patients were classified as chronically infected when, in any of the years during the observation period, the above criteria were met. Since our center does not test for *A. xylosoxidans* specific antibodies, only criteria “a” above was considered.

We defined a “single infection” as one in which incidental sputum culture was positive during the entire observation period. We defined “intermittent infection” as having 2 or more positive cultures for *A. xylosoxidans* during the entire observation period but not meeting the above criteria of “chronic infection”, that is, 2 or more positive cultures recurring during the observation period, with months and years of negative cultures in between. Patients who met the “chronic infection” criteria during any given year remained classified as such for the purpose of the analysis.

### 2.3. CFTR Genotype

Using the standard classification of CFTR mutations [18], patients were classified as having “minimal CFTR function” if they had 2 mutations from classes I, II, or III; or “residual CFTR function” if they had at least one mutation from class IV–V.

### 2.4. Sputum Cultures

As part of the routine clinic protocol, patients with CF were seen at 1–6 monthly intervals, usually every 3 months, as well as during periods of clinical deterioration. During each visit, sputum for culture was either expectorated or induced as previously described [19] and was transferred immediately to the department of microbiology.

Sputum specimens were processed following the recommendations of the Clinical Microbiology Procedures Handbook [20]. Gram staining was performed for all samples. Then, automated identification and susceptibility testing were performed. Each isolate was identified using the VITEK 2 system (bioMérieux) or the MALDI Biotyper System (Bruker Daltonics Inc., Billerica, MA, USA), according to the manufacturer’s instructions for bacteria identification. Antimicrobial susceptibility profiles of the isolates were determined using the disk diffusion method, E-test, or VITEK II (bioMérieux) as needed and according to CLSI criteria. Of note, microbiology laboratory techniques have not significantly changed during the observation period with regard to *A. xylosoxidans* identification.

We calculated the absolute change in FEV_1_ after *A. xylosoxidans* acquisition by taking the FEV_1_ percent predicted measured as close as possible to the acquisition date, and the FEV_1_ percent predicted, measured as close as possible to 1 year and 2 years after acquisition, and calculating the difference between them. We calculated pulmonary exacerbations by documenting the number of intravenous antibiotic courses.

### 2.5. Statistical Analysis

Patient demographic and clinical characteristics were summarized using median and interquartile range, or mean and standard deviation, as appropriate. Proportions were calculated for categorical variables. Parameters were compared between *A. xylosoxidans* positive and negative subjects using paired student’s *t*-tests, or chi-squared test, as appropriate. Linear regression analysis was performed to test for associations between *P. aeruginosa* infection and pulmonary outcomes in *A. xylosoxidans*-infected subjects. All analyses were 2-tailed, and a *p*-value < 0.05 was considered significant.

### 2.6. Ethics Board Approval

This study was approved by the local ethics Institution Review Board (IRB), RMC-0284-20 on 12 June 2020. This study is a retrospective analysis based on de-identified data extracted from patients’ medical records. As such, the IRB granted an exemption from the requirement for obtaining informed consent.

## 3. Results

During the 14-year observation period, an annual average of 124 patients were followed at our clinic, gradually increasing over time, from 86 in 2007 to 148 in 2020. The observation period occurred before the state approval and widespread usage of highly effective CFTR modulators.

Table 1 displays the demographic and clinical characteristics of pwCF who were ever positive for *A. xylosoxidans* versus those who were never positive. With the exception of FEV_1_%pp, which was lower in the *A. xylosoxidans*-positive group, there were no differences, although patients with *A. xylosoxidans* tended to have a higher proportion of pancreatic insufficiency, CF-related diabetes, and chronic *P. aeruginosa* infection, without reaching statistical significance.

### 3.1. A. xylosoxidans Prevalence and Chronicity

**Figure 1:**
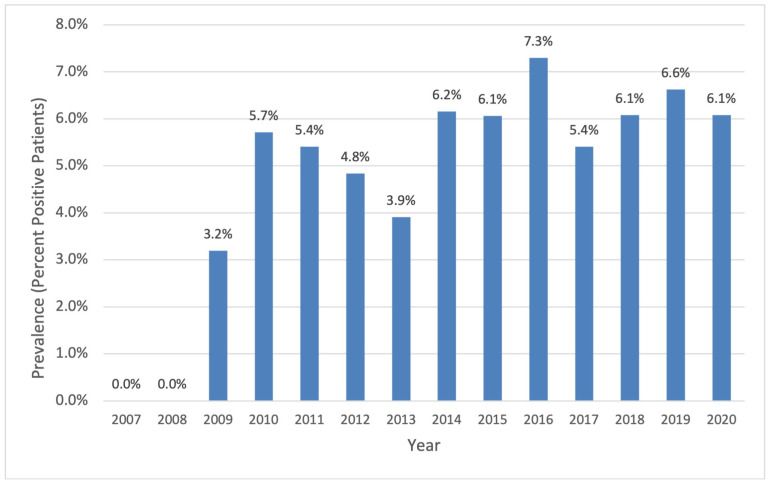
Percent of patients with CF with any *A. xylosoxidans*-positive sputum culture (single, intermittent, and chronic) of total CF clinic population per year.

A total of 29 patients of our entire CF cohort had at least one positive culture with *A. xylosoxidans* throughout the study period. During the first two years of data collection (2007–2008), no sputum cultures were positive for *A. xylosoxidans*. Thereafter, a fluctuating increase was observed, stabilizing from 2014 until the end of the observation period, with a mean (SD) annual prevalence of 6.2% (±0.5) during these seven years (Figure 1).

Nine patients (31% of patients ever infected with *A. xylosoxidans*) met the criteria of “chronic infection”, seven (24% of all *A. xylosoxidans* positive patients) had “intermittent infection” and thirteen (45% of patients infected with *A. xylosoxidans*) only one single positive culture during the study period.

### 3.2. Change In Clinical Status Following Acquisition of A. xylosoxidans

The frequency of pulmonary exacerbations requiring a course of IV antibiotics for all *A. xylosoxidans*-infected patients did not differ before versus after acquisition, with a median of two courses (range 0–9) in the two years before, compared to a median of two courses (range 0–9) in the two years after acquisition, *p* = 0.38. However, subgroup analysis demonstrates that patients with intermittent and chronic infection taken together suffered more exacerbations post-acquisition, with a median (range) 2.5 (0–8) exacerbations pre- and 3 (0–9) post-acquisition (*p* = 0.036). Patients with a single sporadic infection had a median of 2 (0–9) exacerbations pre and 1 (1–8) post-acquisition, *p* = 0.10.

**Figure 2:**
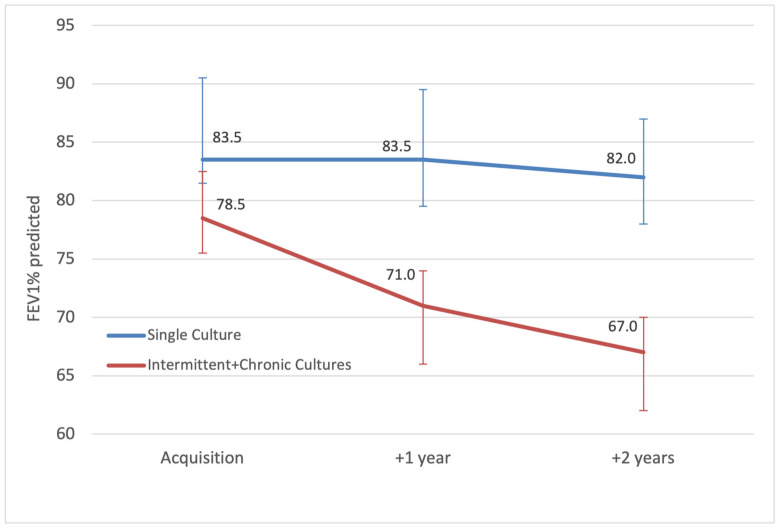
Median annual FEV_1_ percent predicted (error bars showing IQR) of patients with intermittent and chronic *A. xylosoxidans*, compared to patients with a single culture, from acquisition until 2 years after.

When comparing the lung function of all pwCF ever infected with *A. xylosoxidans* versus all negative patients, there was no excessive deterioration in FEV_1_% at 1 year (−0.2%) or 2 years (−0.6%) post-acquisition, *p* = 0.83 and *p* = 0.25, respectively. However, subgroup analysis demonstrates that chronically and intermittently infected patients taken together had a more significant median FEV1% decline of 11.5% (IQR = −3.5–7.5) at 2 years post-acquisition, compared to a median decline of only 1.5% (IQR = −2.5–12.5) for patients with a single positive culture, *p* = 0.03 (Figure 2).

After *A. xylosoxidans* acquisition, there was no statistically significant BMI decline, from 20.1 kg/m^2^ at baseline to 20.5 kg/m^2^ at 1 year and at 20 kg/m^2^ at 2 years post-acquisition, *p* = 0.10 and *p* = 0.13, respectively. The mean BMI of patients without *A. xylosoxidans* also remained stable during this period, at 20.4 kg/m^2^, *p* = NS. Subgroup analysis comparing intermittently and chronically infected patients taken together versus patients with a single infection was also non-significant.

No patient with *A. xylosoxidans* underwent a lung transplant. One patient with *A. xylosoxidans* died during the observation period. This patient had a single isolation, and his demise was deemed unrelated to the *A. xylosoxidans* infection.

### 3.3. The Effect of Concurrent P. aeruginosa Infection

Chronic *A. xylosoxidans* infection was not associated with chronic *P. aeruginosa* infection (*p* = 0.46). Linear regression analyses were performed to assess the impact of chronic *P. aeruginosa* infection on the decline in lung function, as measured by FEV_1_.

There was no significant correlation with a decline greater than 4% in one year (*p* = 0.52) or greater than 5% in two years (*p* = 0.89). When analyzing the effect of chronic co-infection with *A. xylosoxidans* and *P. aeruginosa*, there was equally no significant association with a decline greater than 4% in one year (*p* = 0.66) or greater than 5% in two years (*p* = 0.53). Among the 29 patients with *A. xylosoxidans* infection, 11 experienced an increased need for IV antibiotic courses in the two years following *A. xylosoxidans* acquisition compared to the two years prior. Notably, 10 of these 11 patients also had chronic *P. aeruginosa* infection. A borderline significant positive association was observed between *P. aeruginosa* infection and the need for IV treatment (*p* = 0.052).

### 3.4. Antibiotic Susceptibility

The antibiogram results of all 296 sputum cultures collected during the observation period demonstrated very high in vitro sensitivity to beta-lactams, including Carbapenems (especially Ertapenem and Imipenem), Minocycline, and extended-spectrum Penicillins (Piperacillin, Piperacillin–Tazobactam, Amoxicillin–Clavulonate), and low susceptibility to Cephalosporins evaluated. Antibiogram results are presented in Table 2.

## 4. Discussion

### 4.1. Association of A. xylosoxidans with More Severe Lung Disease

In this single-center study spanning a 14-year observation period, patients with *A. xylosoxidans* infection appeared to have more severe disease at baseline, as evident from lower FEV_1_, as well as a tendency to present with a higher proportion of pancreatic insufficiency, CFRD, and chronic *Pseudomonas* infection. A similarly designed French retrospective case–control study also found *A. xylosoxidans*-infected patients to have had more frequent pulmonary exacerbations, hospitalizations, and intravenous and oral antibiotic courses over the 2 years prior to acquisition [21]. As opposed to those with a single episode of infection, patients in our cohort with chronic and intermittent infection experienced accelerated lung function decline and an increase in the frequency of pulmonary exacerbations, another finding mirrored by the French study [21].

### 4.2. Prevalence and Age at Acquisition

During our observation period, about one-fifth of all pwCF were infected at least once with *A. xylosoxidans*, in line with reports for pwCF in other centers [12,22]. The prevalence increased with fluctuations during the first half and appeared to have reached a plateau during the second half. The initial rise in prevalence may be due to patient-to-patient spread [23,24]. Due to the lack of genotyping in our laboratory, we can only speculate that the observed plateau in *A. xylosoxidans* prevalence in later years may be attributed to more rigorous isolation practices”.

The median age at acquisition was late adolescence, which was also shown by others [3,4,6,14,25] and similar to the age of acquisition of other emerging CF bacteria, such a non-tuberculous mycobacteria [26]. The predilection of these pathogens for the CF lung with more advanced disease may be explained by favorable metabolic niche conditions, such as thick mucus, nutrient availability, altered oxygen levels, chronic inflammation, biofilm formation, and an impaired immune response [12].

### 4.3. European Cystic Fibroses Society Registry Data

Kerem et al. recently examined the European CF Society Patient Registry data for cross-sectional demographic and clinical characteristics, as well as outcomes associated with *Achromobacter* species [16]. In a total of 38,795 eligible pwCF, *Achromobacter* infection was associated with disease severity similar to infection with *Pseudomonas aeruginosa*. Being infected with both bacteria was associated with even more severe disease. Despite the inherent limitations of this registry study, such as its cross-sectional design, variable data quality and consistency, and potential selection bias, the examination of such a large patient cohort provides additional support for the hypothesis that *Achromobacter* may indeed contribute to disease severity rather than just serve as a surrogate marker. Our data, from a smaller sample but with the benefit of longitudinal follow-up and more granularity concerning the frequency of bacterial growths, are in line with the registry findings. Definitive claims regarding this relationship would require evidence from different types of trial designs, such as randomized controlled trials, which may not be considered ethical given the current state of evidence.

### 4.4. Possible Confounding Through Co-Infection with P. aeruginosa

*Achromobacter species* and *P. aeruginosa* exhibit several pathophysiological similarities in the context of CF, which contribute to their persistence in the CF lung. These include their capability to produce biofilms [27], complex resistance mechanisms [28], and their possession of various virulence factors that enhance their pathogenicity [29]. We examined whether co-infection with *P. aeruginosa* may indicate more severe disease. No accelerated decline in lung function was observed with either isolated chronic *P. aeruginosa* infection or in combination with *Achromobacter*. Sample size constraints might account for the failure to demonstrate this. Another possible explanation is the aggressive treatment and eradication protocols implemented upon detection of *P. aeruginosa*, which may mitigate its impact on lung function. Conversely, *Achromobacter*, which does not always prompt intensive antibiotic treatment upon isolation, might exert a more subtle effect that is not readily captured in short-term lung function analyses. The borderline significant association between *P. aeruginosa* and the increased need for IV antibiotics suggests a possible link; however, as IV therapy is also used for eradication purposes—even in the absence of clinical exacerbation—this association should be interpreted with caution.

### 4.5. Antibiotic Resistance Patterns

In our cohort, sputum cultures demonstrated high sensitivity of *A. xylosoxidans* to Carbapenems (especially Ertapenem and Imipenem) and high sensitivity to Minocycline, a tetracycline antibiotic. This is in contrast to other reports in which only about 50% of isolates were sensitive to Minocycline [10]. Piperacillin, Piperacillin–Tazobactam, and Amoxicillin–Clavulonate also demonstrated very high sensitivity against *A. xylosoxidans*. Conversely, as expected, the lowest in vitro sensitivity was found for Aminoglycosides and quinolones. None of our *A. xylosoxidans* strains exhibited particular antibiotic resistance.

### 4.6. Study Limitations

Our study is limited by its retrospective design and a relatively small sample size. To compare culture-positive patients with those who are culture-negative, an arbitrary cohort had to be chosen. We elected to use data from our CF clinic database, collected in 2014, which is the midpoint of our observation period (excluding all patients who were ever positive for *Achromobacter*). We are aware that this comparison is artificial and may have skewed the results; however, it was primarily used for basic demographic and clinical features. Regarding highly effective CFTR modulators, these were introduced only toward the end of our study period and in a limited number of patients. As a result, isolating their impact on *A. xylosoxidans* prevalence and associated morbidity was neither feasible nor within the scope of the study.

## 5. Conclusions

The present study contributes to the growing body of evidence regarding the increasing prevalence and emerging pathogenicity of *A. xylosoxidans* in CF. Intermittent and chronic infection had a detrimental influence on lung function and pulmonary exacerbations. Despite the inability to demonstrate causality, we infer that it may be reasonable to attempt eradication whilst awaiting larger prospective longitudinal studies to confirm our findings. Future research ought to better characterize strain dynamics and transmission patterns, in particular considering the effect of highly effective CFTR modulators on the prevalence and clinical impact of *A. xylosoxidans*.

## Figures and Tables

**Table 1:** Demographic and clinical characteristics of patients ever positive for *A. xylosoxidans* versus patients never positive (*n* = 124).

	*A. xylosoxidans* Positive * (*n* = 29)	*A. xylosoxidans* Negative ** (*n* = 95)	*p*-Value
Male, *n* (%)	14 (48%)	54 (43%)	0.7
Age (years), median (IQR)	17 (14.5–33)	13.6 (7.6–27.7)	0.13
Minimal CFTR function genotype *n* (%)	19 (66%)	46 (48%)	0.15
Pancreatic Insufficiency *n* (%)	19 (66%)	45 (47%)	0.07
CF-Related Diabetes *n* (%)	5 (17%)	9 (7%)	0.09
FEV_1_% median (IQR)	81 (46–94)	90 (72–99)	<0.001
BMI, mean (SD)	20.1 (4.1)	20.3 (4.6)	0.86
Chronic *Pseudomonas aeruginosa* *** *n* (%)	16 (55%)	34 (27%)	0.06

* Data retrieved at time of acquisition; ** data retrieved from CF clinic database 2014 (mid-point of observation period); *** >50% positive cultures in preceding 12 months.

**Table 2:** Antibiogram of all *Achromobacter xylosoxidans* cultures, showing the percentage of cultures sensitive, resistant, or intermediate to the respective antibiotics tested.

	%Sensitive	%Resistant	%Intermediate
Ertapenem	100%	0%	0%
Piperacillin	97.4%	2.6%	0%
Minocycline	96.6%	3.4%	0%
Piperacillin/Tazobactam	92.6%	3.7%	3.7%
Amoxicillin/Clavulonate	92.5%	5%	2.5%
Imipenem	90.9%	5.7%	3.4%
Ceftazidime	86.7%	5.3%	8%
Meropenem	70.3%	26.6%	3.2%
Sulfamethoxazole/Trimethoprim	54.6%	43.5%	1.9%
Colistin	34.1%	41.5%	24.4%
Ofloxacin	23.1%	72.3%	4.6%
Ampicillin	12.5%	62.5%	25%
Ciprofloxacin	9.7%	70.9%	19.4%
Tobramycin	8.2%	88.5%	3.3%
Gentamicin	6.2%	91.4%	2.5%
Amikacin	4.9%	91.4%	3.7%
Cefuroxime	2.4%	97.6%	0%
Cephalothin	0%	100%	0%

## Data Availability

The original contributions presented in this study are included in the article. Further inquiries can be directed to the corresponding author.
